# Reproducibility of multiple T1 Mapping techniques and to ECV quantification

**DOI:** 10.1186/1532-429X-16-S1-P18

**Published:** 2014-01-16

**Authors:** Teerapat Yingchoncharoen, Chirstine Jellis, Zoran Popovic, Scott Flamm, Deborah Kwon

**Affiliations:** 1 Cleveland Clinic, Cleveland, Ohio, USA

## Background

T1 Mapping has emerged as a new marker to quantify myocardial extracellular volume (ECV), which is typically increased in the setting of diffuse fibrosis or infiltrative heart diseases. However the reproducibility of the technique is largely unknown. We sought to identify the interobserver, intraobserver variability of different T1 mapping techniques as well as validation of one technique against the standard technique for ECV quantification.

## Methods

We selected 10 patients with the diagnosis of cardiomyopathy (5 with delayed contrast enhancement and 5 without). The Look-Locker (LL) and Modified Look-Locker both precontrast (MOLLI_Pre) and postcontrast (MOLLI_Post) T1 time were measured by 3 investigators blinded to clinical data at 2 different time points. Interobserver and Intraobserver reproducibility were assessed using 2-way ANOVA approach. The ECV was calculated using pre and post contrast T1 and hematocrit.

## Results

There was a strong correlation between LL T1 and Post-contrast MOLLI(MOLLI_Post) (R2 0.82, p < 0.001) and a modest correlation between MOLLI_post and ECV (R2 = 0.42, p < 0.001). The interobserver and intraobserver variability of the measurements were expressed as standard errors of measurements (SEM) and 95% CI and were summarized in table 1. MOLLI_Post had the lowest intraobserver and intraobserver variability with minimal detectable change in T1 of 13.8 and 26.1 respectively. ECV showed minimal both interobserver and intraobserver variability.

**Table 1:** Interobserver and intraobserver variability of LL, MOLLI_Pre, MOLLI_Post and ECV

	LL	MOLLI_Pre	MOLLI_Post	ECV
Intraobserver	5.7	14.5	5.0	0.9

Min Δ detectable	15.7	40.1	13.8	2.6

Interobserver	29.1	15.7	9.1	1.9

Min Δ detectable	80.7	43.5	26.1	5.3

## Conclusions

Both LL and MOLLI are highly reproducible technique. LL was strongly correlated with post-contrast MOLLI which is moderately correlated to ECV.

## Funding

None.

